# Predicting postoperative motor outcomes in the surgical management of Rolandic focal cortical dysplasia: the role of glucose metabolism

**DOI:** 10.1186/s12916-025-04213-9

**Published:** 2025-07-01

**Authors:** Weiyuan Luo, Bowen Yang, Xiaobin Zhao, Baotian Zhao, Chao Zhang, Xiu Wang, Jiajie Mo, Xiangzhi Lu, Zhong Zheng, Xiaoqiu Shao, Jianguo Zhang, Kai Zhang, Wenhan Hu

**Affiliations:** 1 Beijing Neurosurgical Institute, Capital Medical University, Beijing, China; 2 Department of Neurosurgery, Beijing Tiantan Hospital, Capital Medical University, Beijing, China; 3 Department of Nuclear Medicine, Beijing Tiantan Hospital, Capital Medical University, Beijing, China; 4 Department of Neurosurgery, Beijing Fengtai Hospital, Beijing, China; 5 Department of Neurology, Beijing Tiantan Hospital, Capital Medical University, Beijing, China; 6 Beijing Key Laboratory of Neurostimulation, Fengtai District, No.119 South 4 Ring West Road, Beijing, China

**Keywords:** Rolandic epilepsy surgery, [^18^F]FDG PET, Motor function, Functional reorganization, Focal cortical dysplasia

## Abstract

**Background:**

This study aimed to explore the predictive value of ^18^F-fluorodeoxyglucose positron emission tomography ([^18^F]FDG PET) metabolic activity in determining postoperative motor function outcomes in patients with Rolandic focal cortical dysplasia (FCD).

**Methods:**

We conducted a retrospective analysis of 62 patients with FCD who underwent resective surgery in the Rolandic area. Of these, 15 patients underwent task-based functional magnetic resonance imaging (fMRI). Motor functional reorganization and its relationship with PET metabolism were analyzed. Patients were classified into deficit and non-deficit groups according to their postoperative motor function status. PET metabolism in the resected motor cortex was compared between the two groups, and its correlation with postoperative muscle strength was further evaluated in the deficit group.

**Results:**

Among the 15 patients who underwent fMRI evaluation, the extent of motor reorganization was negatively correlated with PET metabolism (*P* = 0.0006). PET analysis revealed significantly higher PET metabolic *T*-values in the resected Rolandic cortex of the motor deficit group compared to the non-deficit group (*P* = 0.0004). Furthermore, within the deficit group, PET metabolic *T*-values were negatively correlated with postoperative muscle strength (*P* = 0.017). The prediction model for postoperative motor function, demonstrated strong performance in cross-validation, with an area under the curve (AUC) of 0.82, a sensitivity of 83%, and a specificity of 76%.

**Conclusions:**

The findings underscore the critical role of preoperative [^18^F]FDG-PET in predicting motor outcomes following Rolandic cortex resection in epilepsy patients, highlighting its value in optimizing preoperative assessment and surgical planning.

**Supplementary Information:**

The online version contains supplementary material available at 10.1186/s12916-025-04213-9.

## Background

Resective surgery may be considered for epilepsy patients who continue to experience uncontrolled seizures despite optimal pharmacotherapy [1, 2]. In such cases, the primary objectives of resective surgery include the complete resection or disconnection of the epileptogenic zone (EZ) while preserving the eloquent cortex [3, 4]. The Rolandic cortex, responsible for contralateral motor and sensory functions, is a common site for focal cortical dysplasia (FCD) [3, 5], making it a frequently targeted region in epilepsy surgery. Numerous studies have focused on delineating motor functional areas preoperatively and protecting motor functions during surgery. These efforts encompass preoperative functional MRI (fMRI) [6], cortical mapping [7], magnetoencephalography (MEG) [8], and intraoperative techniques such as neuro-navigation [9], motor evoked potentials (MEP) [10], and awake craniotomy [11]. Despite the advent of these sophisticated techniques, they operate independently of preoperative epilepsy evaluation data and often require additional procedures, some of which necessitate patient cooperation, making them unsuitable for pediatric patients. Extracting motor function localization data from preoperative epilepsy assessments would represent a significant advancement and offer substantial benefits to patients.

Positron emission tomography with ^18^F-fluorodeoxyglucose positron emission tomography ([^18^F]FDG PET), a functional neuroimaging modality, plays a crucial role in localizing epileptic foci and has become a routine component of preoperative assessment in most comprehensive epilepsy centers [12–14]. Theoretically, [^18^F]FDG PET imaging detects glucose metabolism in brain tissues, thereby reflecting the functional state of a region. In the context of EZ localization, most EZs exhibit dysfunction during interictal periods, manifesting as hypometabolism on [^18^F]FDG PET images. According to the five-zone model proposed by Lüders [15], the hypometabolic region identified through PET imaging corresponds to the functional deficit zone, which may overlap with or be adjacent to the EZ. The term “function” in this context refers to normal neurophysiological processes, including motor, sensory, memory, and language abilities. Previous studies on temporal lobe epilepsy have demonstrated that PET hypometabolism in specific brain regions is associated with impairments in memory, cognition, and language functions [16–18]. However, to date, no studies have examined the relationship between glucose metabolism and motor functions in the Rolandic cortex.

In this study, we will focus on the pathological substrate of FCD and utilize [^18^F]FDG PET imaging to investigate the impact of such epileptic foci on motor functions within the Rolandic cortex. FCD was selected as the subject of our research because, compared to other epileptogenic lesions such as tumors, vascular malformations, and ulegyria, it most closely resembles normal cerebral cortex. It is characterized by disruptions in cortical architecture and the presence of abnormal neurons [19]. Beyond the direct effects of epileptic seizures, FCD does not exert additional impacts on the Rolandic cortex, such as tumor-induced compression or hemorrhage from vascular malformations. We believe this study will provide valuable imaging evidence for the preoperative localization of motor functional areas in Rolandic epilepsy, thereby improving postoperative motor function outcomes for patients.

## Methods

### Patient selection

In this study, we retrospectively included patients who underwent resective epilepsy surgery for drug-resistant FCD in the Rolandic area at the Epilepsy Center of Beijing Tiantan Hospital and Beijing Fengtai Hospital between September 2015 and December 2023. All patients included in the study underwent a comprehensive preoperative evaluation, including clinical history, neurological examination, scalp electroencephalography (EEG) monitoring, routine MRI, [^18^F]FDG PET, and image post-processing (including PET/MRI co-registration techniques). In addition, a subgroup of subjects underwent fMRI scanning, which was introduced during the mid-to-late stage of the study period, beginning in 2020. The extent of the EZ was determined through a comprehensive presurgical evaluation involving input from at least two experienced epilepsy specialists. The following criteria were used to determine patient inclusion in this study: (1) the EZ was localized in the Rolandic area; (2) pathology was confirmed as FCD; (3) all patients had normal preoperative muscle strength (grade 5); and (4) none of the patients had undergone previous brain surgery. To analyze the relationship between postoperative motor function outcomes and PET metabolism, patients were divided into deficit and non-deficit groups based on whether they had recovered to their preoperative status within 1 week after surgery. Additionally, patients in the deficit group were further stratified by the degree of distal limb muscle strength for a subgroup analysis [20]. To evaluate the potential impact of patient characteristics, such as age, gender, duration, and pathological classification, on postoperative motor function, we conducted additional statistical analyses.

### Resection volume and classical motor functional area acquisition

Experienced neurosurgeons assessed the resection volume through manual contour segmentation using MRIcron (https://www.nitrc.org/projects/mricron), delineating the resection extent based on postoperative CT or MRI scans in native space. Each individual resection volume map was then normalized to MNI standard space using deformation fields obtained through SPM12 (https://www.fil.ion.ucl.ac.uk/spm/software/spm12/). The acquisition of classical motor functional areas was divided into two parts: (1) the Rolandic area was precisely extracted and delineated based on segmentation of the AAL atlas, performed using MATLAB 2022b (MathWorks); and (2) the classical motor cortex areas, including the hand-knob and paracentral lobule, were assessed by experienced neurosurgeons to evaluate the location of functional areas activated in the fMRI of 60 normal controls. These areas were then manually outlined on the AAL atlas using MRIcron, resulting in the identification of four distinct regions corresponding to the bilateral hand-knob and paracentral lobule [21].

### *[*^*18*^*F]FDG PET acquisition and PET/MRI co-registration*

The [^18^F]FDG PET examination was conducted using a GE Discovery ST PET-CT system (300 mm FOV, matrix 192 × 192, 3.27-mm slice thickness). An intravenous injection of [^18^F]DG at a mean dose of 220 MBq/70 kg body weight was administered. No patients exhibited an ictal event less than 6 h prior to or during the [^18^F]FDG PET scan. Given the inherent limitations of PET imaging, particularly in terms of resolution and precise anatomical localization of gray and white matter, the integration of PET and MRI images through coordinate matching and fusion reading offers a valuable method for clinicians to visualize hypometabolism in gray matter and identify the EZ. SPM12, running on MATLAB 2022b (MathWorks), was used for the co-registration and analysis of the [^18^F]FDG PET images [22].

### PET analysis

For the quantitative analysis of [^18^F]FDG PET images, the data were preprocessed using spatial normalization and signal-to-noise ratio enhancement with a Gaussian smoothing kernel with a full-width at half-maximum (FWHM) of 8 mm. Following preprocessing, the images of each subject were compared with those of 52 control subjects without neurological disease who had negative [^18^F]FDG PET reports [23]. A voxel-based independent two-sample *t*-test was then conducted, with age and gender as covariates. Prior to the *t*-test, each individual’s PET images were normalized by dividing them by their whole-brain mean FDG uptake value to control for individual differences, as detailed in our previous studies [24, 25]. Finally, an unthresholded SPM-PET T-score map was generated, representing the relative quantitative metabolic change in comparison with the control group for each individual [14]. For the analysis of patients’ postoperative motor function, we assessed the overlap between each patient’s surgical resection volume and the Rolandic area. The overlapping regions were extracted from each SPM-PET T-score map, and the mean *T*-value of these regions was used to represent metabolic activity. This approach enabled us to investigate the relationship between postoperative motor function and PET metabolism in patients whose surgical resections involved the Rolandic cortex. A schematic representation of the PET analysis workflow has been included in the Additional file 1: Fig. S1 to provide a clearer overview of the process.

### fMRI acquisition

Task-based fMRI was performed to investigate the reorganization of motor functional regions in Rolandic FCD and its correlation with PET metabolism. Given the critical role of the Rolandic region in motor control, fMRI was particularly recommended for patients with lesions near or involving the precentral gyrus, where precise localization of motor areas is essential for surgical planning and functional preservation. Additionally, in cases of suspected functional reorganization—especially in patients with long-standing epilepsy or early-onset seizures—fMRI was used to assess potential shifts in motor representations due to cortical plasticity. fMRI was also integrated with diffusion tensor imaging (DTI) to evaluate the relationship between functional activation and the underlying white matter pathways, particularly in cases where lesions were adjacent to the corticospinal tract. For certain patients, especially young children, task-based fMRI examinations may not be feasible due to their inability to cooperate with task performance, and as a result, they were excluded from such assessments.

All fMRI data were acquired on a 3T Siemens MR scanner. Subjects were instructed to minimize head movement, relax with their eyes closed, and remain awake throughout scanning to reduce motion artifacts and ensure optimal data quality. BOLD images were acquired using an echo-planar imaging sequence with the following parameters: repetition time (TR) = 3400 ms, echo time (TE) = 70 ms, flip angle = 90°, field of view (FOV) = 192 × 192 mm^2^, voxel size = 3 × 3 × 3 mm^3^, and 100 slices. The block-design paradigm consisted of alternating 30-s active and rest periods, repeated over 5 cycles, enabling robust detection of task-related activations and lasting a total of 5 min. For upper limb function, participants performed repetitive, self-paced thumb-to-finger opposition, while for lower limb function, they engaged in active ankle dorsiflexion and plantarflexion. This design aimed to localize motor activation in both the upper and lower extremities.

### fMRI analysis

Preprocessing of fMRI data included converting images from DICOM to NIfTI format using MRIcron. T1-weighted images were segmented to extract gray and white matter, which were then used to generate a binary mask for skull stripping. Slice timing correction (TR = 2 s, TA = 1.97 s, interleaved slice order) was performed, followed by spatial realignment (quality = 0.9, separation = 4 mm). Coregistration to T1-weighted images was conducted using SPM12, followed by spatial normalization to MNI space to ensure cross-subject alignment and enhance spatial consistency. To improve the signal-to-noise ratio, 3D Gaussian smoothing with an isotropic Gaussian kernel (FWHM = 4 mm) was applied. Stimulus onset times for hand and foot movements were predefined, with each task lasting 30 s. A high-pass filter (128 s) was applied to eliminate low-frequency noise, and an autoregressive model was used to correct for temporal auto-correlations. Voxel-wise *t*-tests with family-wise error correction (*P* < 0.05) were performed to identify significant activations [26], generating individual-level functional activation maps that provided insights into task-related neural activity for each subject. Functional activation maps were normalized to MNI space, and overlap rates with bilateral hand-knob and paracentral lobule ROIs were calculated to evaluate functional reorganization. Specifically, PET metabolic values were extracted from these classical motor ROIs to investigate their correlation with the calculated overlap rates. This method provided an objective assessment of the relationship between the degree of functional reorganization (as reflected by the overlap rates) and regional cortical metabolism within the classical motor cortex. Correlations with PET metabolism were analyzed using Pearson’s correlation. All analyses were performed using MATLAB 2022b (MathWorks) with SPM12.

## Statistical analysis

The relationship between postoperative motor deficits and PET metabolic *T*-values was assessed using the Mann–Whitney *U* test to compare PET metabolic T-values between the deficit and non-deficit groups. Among patients with motor deficits, the association between muscle strength grades and SPM-PET T-scores was further analyzed using the Kruskal–Wallis test. Additionally, the correlation between the functional activation overlap rate and SPM-PET *T*-scores was evaluated using Pearson’s correlation.

To assess the predictive performance of PET analysis, patients were stratified based on the presence of motor deficits. The mean *T*-values from the overlap between the resected volume and the Rolandic area on the SPM-PET *T*-score map for each patient were randomly assigned to training and testing datasets. A five-fold cross-validation approach was employed, with the training set used for model development and the test set used to evaluate model accuracy. Univariate logistic regression model was constructed, with results reported as odds ratios (OR) and 95% confidence intervals (95% CI). Statistical significance was defined as a two-sided *P*-value < 0.05. Sensitivity, specificity, and the area under the curve (AUC) were also calculated to further assess the model’s performance. All statistical analyses and modeling were performed using R software version 4.2.0.

## Results

### Patients’ characteristics

In this study, 62 patients (29 females, 46.8%) were included, all of whom underwent epilepsy surgery involving resection of the Rolandic cortex. Postoperative pathology confirmed the diagnosis of FCD in all cases. Notably, 41 (66.1%) patients were MRI negative; however, 60 (96.8%) patients exhibited significant hypometabolism within the EZ on PET scans. The mean age at surgery was 15.4 ± 10.3 years, with a mean age of onset of 5.8 ± 4.9 years, and an epilepsy duration of 9.3 ± 9.4 years. The mean follow-up period was 3.6 ± 1.9 years. The lesion laterality distribution was 43.6% left-sided (*n* = 27) and 56.5% right-sided (*n* = 35). According to the Engel classification at the last follow-up, 51 patients (82.3%) were classified as Class I, 9 patients (14.5%) as Class II, and 2 patients (3.2%) as Class III. In a subsequent subgroup analysis, no statistically significant differences were observed between the non-deficit group (*n* = 36) and the deficit group (*n* = 26) across various variables, including age (*P* = 0.38, Mann–Whitney* U* test), gender (*P* > 0.99, Fisher’s exact test), duration of epilepsy (*P* = 0.59, Mann–Whitney *U* test), age of onset (*P* = 0.52, Mann–Whitney *U* test), follow-up duration (*P* = 0.95, Mann–Whitney *U* test), outcome (*P* = 0.90, Mann–Whitney *U* test), laterality (*P* > 0.99, Fisher’s exact test), pathology type (*P* = 0.80, Fisher’s exact test), and MRI manifestation (*P* = 0.90, Fisher’s exact test). The resected volume, including the Rolandic cortex (*P* = 0.12, Mann–Whitney *U* test) and the posterior bank of the precentral gyrus (*P* = 0.10, Mann–Whitney *U* test), also showed no significant differences. Detailed results are presented in Table 1, with specific patient information provided in the Additional file 2: Table S1.

**Table 1:** Demographic and clinical characteristics of the study population

Characteristic	Non-function deficit, *N* = 36	Function deficit, *N* = 26	*P*-value ^a^
Gender F/M	17/19	12/14	> 0.99
Age ^b^ Mean ± SD	16.29 ± 10.54	14.06 ± 9.91	0.38
Duration ^b^ Mean ± SD	8.81 ± 8.87	9.99 ± 10.26	0.59
Onset ^b^ Mean ± SD	5.68 ± 5.66	5.87 ± 3.57	0.52
Follow-up ^b^ Mean ± SD	3.58 ± 1.83	3.62 ± 2.02	0.95
Outcome Engel I A Engel II Engel III	30 5 1	21 4 1	0.90
Lesion side L/R	16/20	11/15	> 0.99
Lesion lobe			0.30
L. FP/Fr/P	1/9/6	3/8/1	
R. FP/Fr/P	0/16/4	1/9/4	
Pathology			0.80
I a/b	1/1	0/1	
II a/b	20/14	16/9	
Resected volume(cm^3^)			
Rolandic cortex	1.99 ± 2.25	3.14 ± 3.08	0.12
PBPG	0.42 ± 0.59	0.77 ± 0.92	0.10
MRI manifestation			0.90
Negative/Positive	23/13	18/8	

^a^ Mann–Whitney *U* test; Fisher’s exact test

^b^ Year

F: female; FP: frontoparietal; Fr: frontal; L: left; M: male; P: parietal; R: right; PBPG: posterior bank of the precentral gyrus

#### Functional reorganization and PET metabolism

**Fig. 1:**
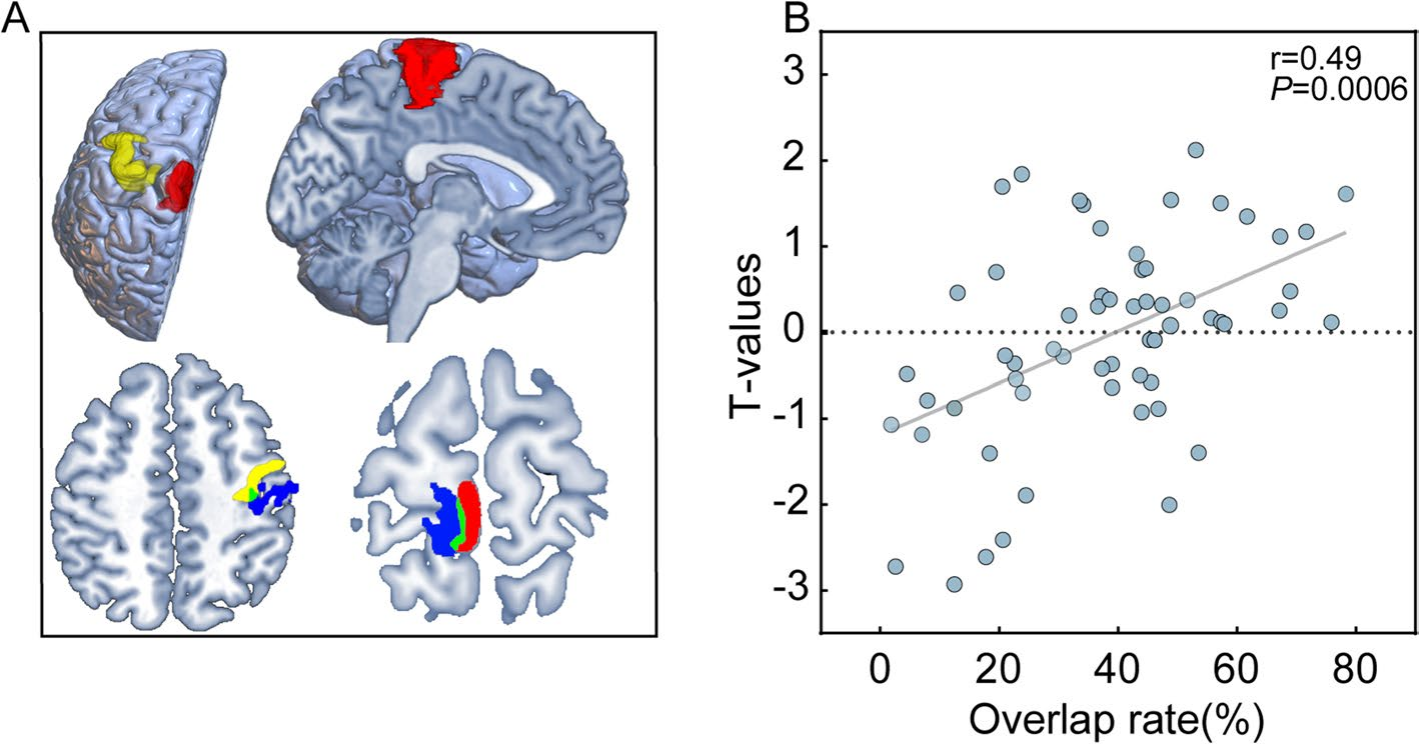
Classical motor cortex ROIs, an example of their overlap with individual functional activation areas, and the correlation between PET metabolic *T*-values and overlap rates are shown. **A** The upper left and upper right panels show the classical motor cortex ROIs, with the yellow region representing the hand-knob area and the red region representing the paracentral lobule. The lower left and right panels illustrate examples of overlap between the classical motor cortex and individual functional activation areas. Yellow and red regions represented the classical motor cortex, blue depicted the patient’s individual activation area, and green indicated the overlap. The left panel showed the overlap for lower hand function, while the right panel demonstrated the overlap for lower limb function. **B** A significant positive correlation was observed between the PET metabolic *T*-values in the classical motor cortex and the overlap rate between the patient’s functional activation area and the classical motor cortex

**Fig. 2:**
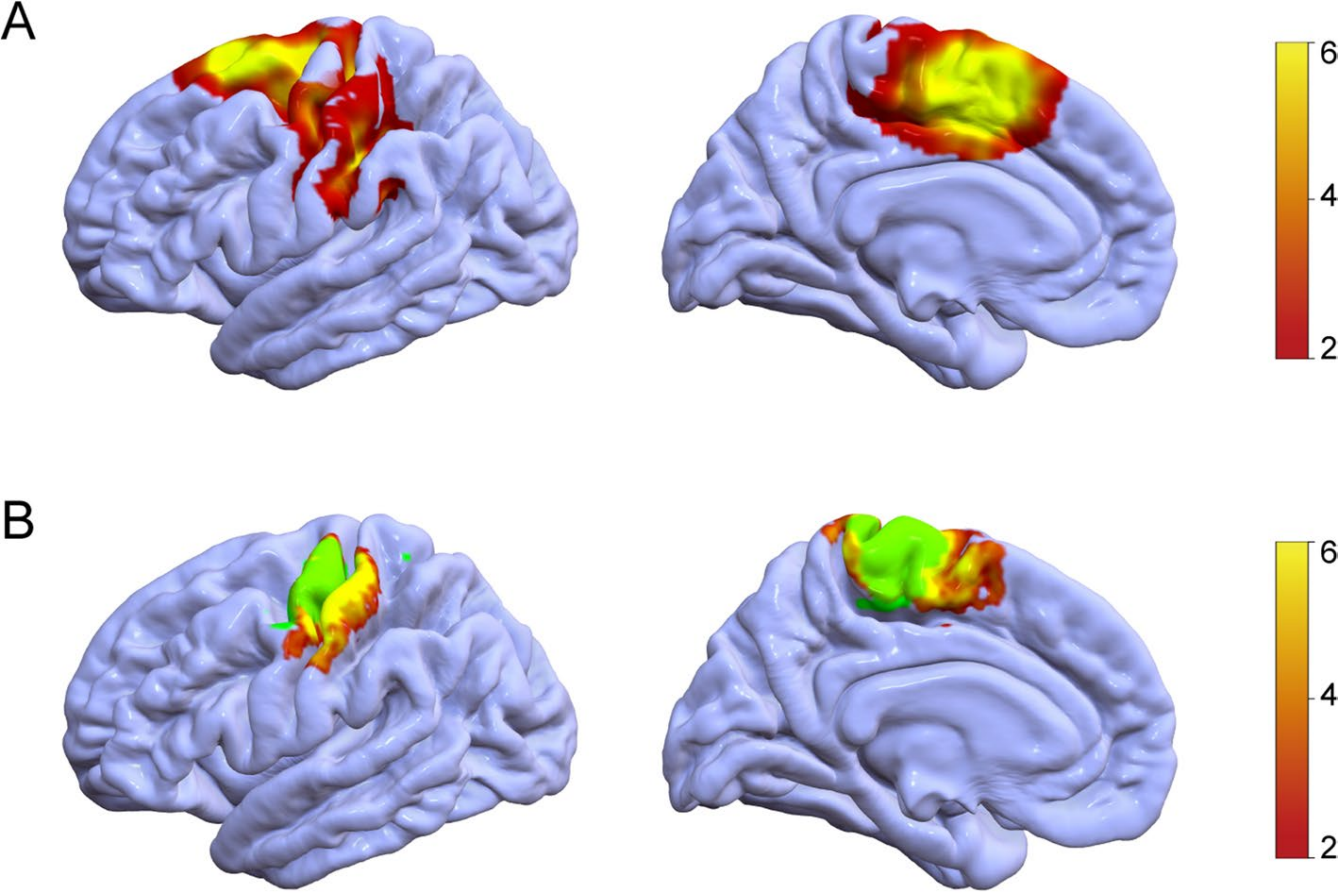
Maps of the EZs and functional activation areas in 15 patients who underwent fMRI. **A** demonstrates the EZs of all 15 patients which were mapped onto the left hemisphere. **B** shows the functional activation areas were mapped onto the cortical surface in the same way, green regions representing the classical functional areas. The left panel illustrates the reorganization of hand motor function, showing a posterior and inferior shift relative to the classical hand-knob region. The right panel depict the reorganization of lower limb motor function, showing an anterior shift toward the paracentral lobule

We included 15 eligible patients who underwent task-based fMRI to investigate the reorganization of motor functional regions in Rolandic cortical FCDs and their correlation with PET metabolism. Figure 1A shows the classical motor cortex ROIs and an example of the overlap rates between individual motor functional activation areas and the classical motor areas. A higher overlap rate suggests minimal functional reorganization, whereas a lower overlap rate indicates significant reorganization, potentially involving functional transfer to other regions. The results demonstrated that the mean overlap rate for the upper limb was 46.96 ± 15.84%, while the mean overlap rate for the lower limb was 46.75 ± 22.16%. A correlation analysis was performed between the overlap rate and PET metabolic *T*-values (*T*-mean) in the classical motor area. Pearson correlation coefficient was *r* = 0.49 (95% CI 0.26–0.66), with *P* = 0.0006 (Fig. 1B). This significant correlation suggests that a higher degree of reorganization in the motor functional area, characterized by reduced motor functional activity in the classical motor area and greater functional transfer to other regions, is associated with decreased PET metabolism in the classical area. We mapped the EZ and functional activation areas of 15 patients onto a standardized brain template (Fig. 2). Among these patients, a posterior and inferior shift in hand motor function activation was observed relative to the classical hand-knob region. Furthermore, functional reorganization of the lower limb was characterized by an anterior displacement of activation within the paracentral lobule (Fig. 2B). Of the 15 patients who underwent preoperative fMRI, 5 (33.3%) developed postoperative motor deficits, compared with 21 of 47 patients (44.7%) without fMRI. Fisher’s exact test showed no significant difference in the incidence of motor deficits between the two groups (*P* = 0.55).

#### Relationships between postoperative motor function and PET metabolism

**Fig. 3:**
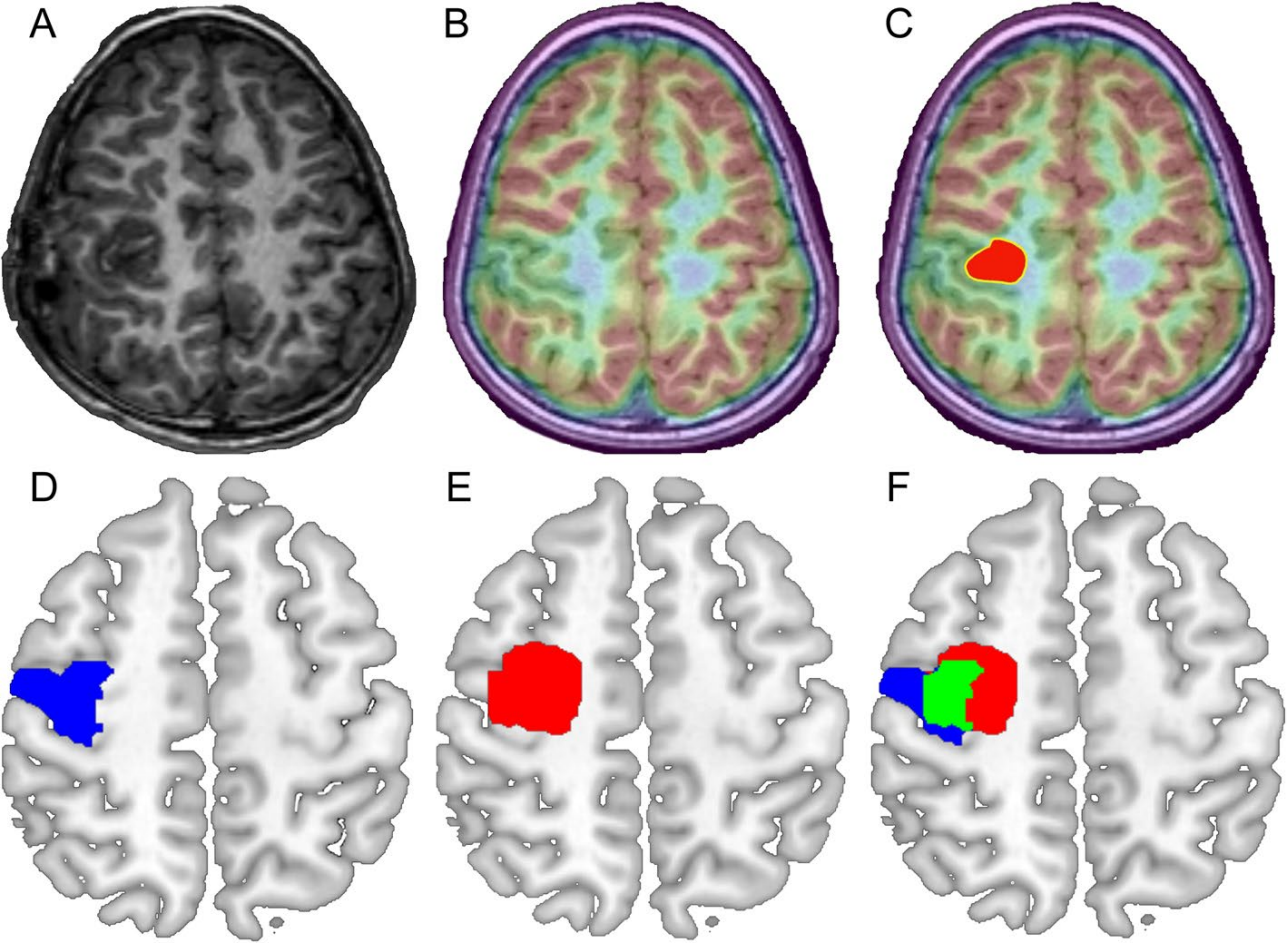
An example of manually delineating a region of interest (ROI) in individual space and its relationship with the Rolandic area after mapping to standard space. **A–C** The process of manually delineating the surgical resection volume in individual space was illustrated. **A** shows the post-surgical axial slice of T1-weighted MRI. **B** is the corresponding FDG-PET/MRI fusion image, significant hypometabolism is visible in the hand-knob. **C** illustrates the surgical resection area (highlighted in red) superimposed on the FDG-PET/MRI fusion image. **D–F** show the overlap between the resection volume and the Rolandic area. **D** shows the Rolandic area in blue. **E** shows the resection area in red. **F** illustrates the overlap between the resection area and the Rolandic area, shown in green

**Fig. 4:**
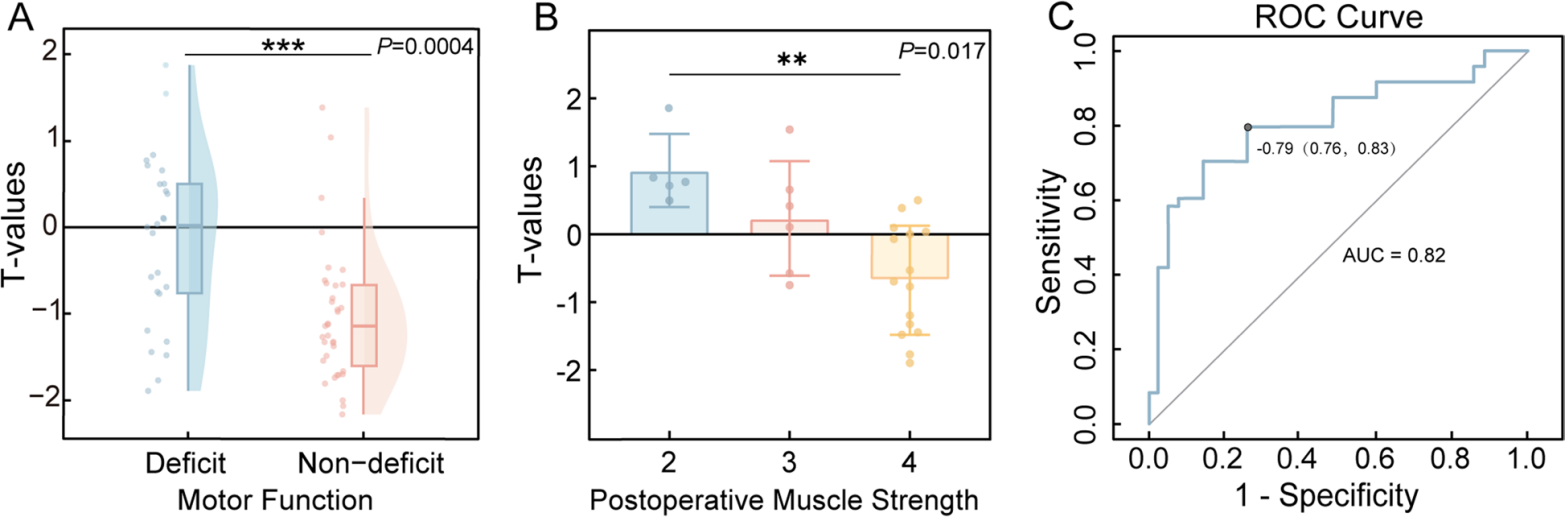
The relationship between PET metabolic *T*-values and postoperative motor function, with the ROC curve of the prediction model. **A** PET metabolic *T*-values in the Rolandic area overlapping the resection region are shown for both the deficit and non-deficit groups. The *T*-values are significantly lower in the non-deficit group compared to the deficit group (*P* = 0.0004). **B** PET metabolic *T*-values in the Rolandic area overlapping the resection region are displayed for patients in the deficit group, categorized by postoperative muscle strength. As muscle strength increased, the *T*-values significantly decreased (*P* = 0.017). **C** Receiver operating characteristic (ROC) curve of the prediction model, demonstrating strong sensitivity and specificity in predicting postoperative motor function based on PET metabolism data

In this study of 62 patients, participants were divided into a motor deficit group (*n* = 26) and a non-deficit group (*n* = 36). Within the motor deficit group, 13 (50%) patients exhibited upper limb muscle strength deficits, 10 (38.5%) patients had lower limb muscle strength deficits, and 3 (11.5%) patients experienced deficits in both upper and lower limbs. For patients with deficits in both upper and lower limbs, the more severe deficit was selected for statistical analysis. Patients were subsequently classified based on postoperative muscle strength grades: 5 patients (19.2%) were graded as 2, 6 patients (23.1%) as 3, and 15 patients (57.7%) as 4. Figure 3 illustrates the surgical resection volume and its corresponding overlap with the Rolandic area. The PET metabolic *T*-values in the resected Rolandic region were significantly lower in the non-deficit group compared to the deficit group (Mann–Whitney *U* test, *P* = 0.0004) (Fig. 4A). Subgroup analyses by histopathological subtype revealed a consistent pattern: PET metabolic *T*-values in the non-deficit group were significantly lower than those in the deficit group for both FCD type IIa (Mann–Whitney *U* test, *P* = 0.0101) and type IIb (Mann–Whitney *U* test, *P* = 0.0158). Further analysis revealed a significant difference in PET metabolic *T*-values among patients with varying muscle strength grades, with PET metabolic *T*-values decreasing as muscle strength grade increased (Kruskal–Wallis test, *P* = 0.017) (Fig. 4B). To evaluate the predictive performance of PET analysis, we developed a logistic regression model using the *T*-value as a predictor and motor deficit as the binary outcome. The model, validated through fivefold cross-validation, demonstrated a sensitivity of 0.83, a specificity of 0.76, and an area under the curve (AUC) of 0.82 (Fig. 4C). The odds ratio (OR) for the association between PET *T*-values and postoperative motor outcomes was 8.67 (95% CI 2.77–25.63), indicating that higher PET metabolic activity within the resected Rolandic cortex significantly increased the risk of postoperative motor impairment. These results provide robust statistical evidence linking PET metabolism with postoperative motor outcomes. To further illustrate this association, Additional file 1: Fig. S2 demonstrates the predicted probability of motor deficits as a function of PET metabolic *T*-values, derived from the logistic regression model across the observed range. The strong correlation between PET metabolic *T*-values and motor function grades, combined with the predictive accuracy of our model, underscores the potential utility of PET metabolism as a reliable marker for identifying motor cortex regions critical to functional preservation.

#### Illustrative case

**Fig. 5:**
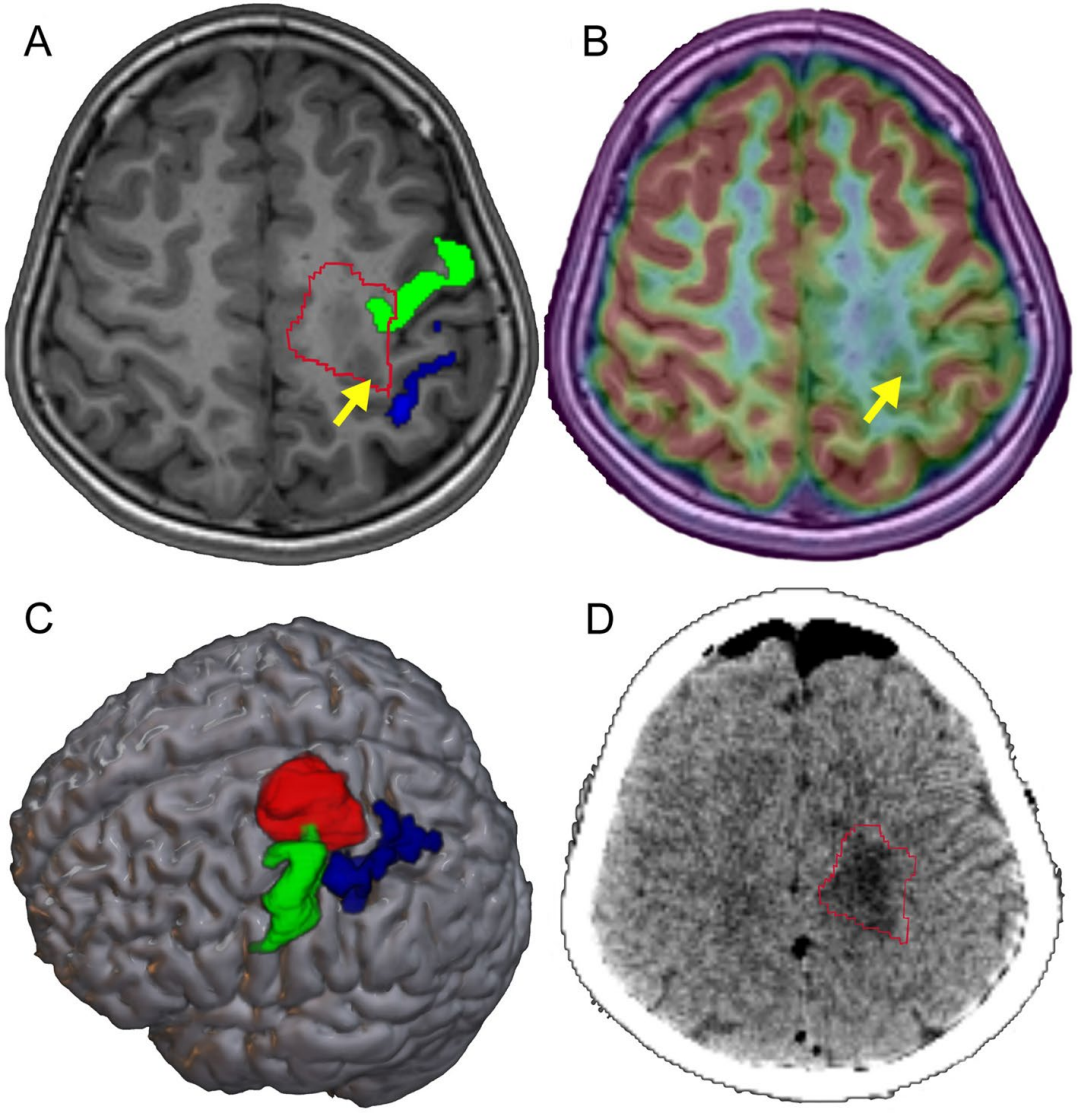
An illustrative case. **A** Preoperative MRI displays the classical hand-knob region (green), the actual hand motor activation area (blue), and the lesion (red). The yellow arrow points to the bottom of the FCD lesion, indicating gray-white matter junction blurring. **B** PET/MRI fusion image shows hypometabolism in the left frontoparietal region. **C** Three-dimensional surface rendering of the patient’s MRI highlights the classical hand-knob region (green), the actual hand motor activation area (blue), and the lesion (red). **D** Postoperative CT illustrates the resected area (red)

An 8-year-old female patient was treated at our center for drug-resistant epilepsy. Despite monotherapy and combination therapies with valproate, oxcarbazepine, levetiracetam, topiramate, and lamotrigine, she continued to experience monthly seizures. Additionally, she suffered seizure clusters every 1.5 years, lasting approximately 2 months, with a daily frequency of 30–40 seizures. Her condition had persisted for 6 years. Seizures were characterized by right-sided limb rigidity, followed by clonic movements. Postictally, she experienced transient right-sided hemiplegia lasting approximately 5–6 min. Preoperative assessment revealed normal muscle strength (grade 5) in all limbs. MRI showed abnormalities in the posterior part of the left superior frontal sulcus, as well as the adjacent precentral and postcentral gyri (Fig. 5A). PET/MRI revealed significant hypometabolism in the left central regions (Fig. 5B). fMRI analysis demonstrated reorganization of the right-hand motor function, which had shifted posteriorly and inferiorly from the classical hand-knob region (Fig. 5A and 5 C). Surgical resection included part of the classical motor area (Fig. 5D), but the patient’s right upper limb strength recovered to grade 4 within 1 week postoperatively. PET imaging showed significantly lower metabolism in the classical motor area compared to the actual functional activation area, supporting our hypothesis that sufficient hypometabolism indicates minimal functional activity in the region. This finding suggests that functional areas may have relocated to more distant regions, thereby reducing the functional impact of resecting classical motor areas.

## Discussion

In this retrospective study, we analyzed epilepsy patients who underwent Rolandic cortical resection with histopathological confirmation of FCD. A strong correlation was identified between the extent of motor function reorganization and PET metabolism, with greater reorganization corresponding to lower PET metabolic levels. Additionally, a significant negative correlation was observed between PET metabolic activity and postoperative motor function. Based on these findings, we developed a predictive model with robust stability and accuracy (AUC = 0.82, sensitivity = 83%, specificity = 76%), which yielded statistically significant results. These findings highlight the potential of preoperative PET metabolic assessment as a valuable tool for predicting motor function outcomes following Rolandic epilepsy surgery, thereby providing critical guidance for both preoperative evaluation and surgical planning.

### The clinical significance of our findings

Resection of the Rolandic cortex remains a significant challenge in epilepsy surgery due to its critical role in motor functions. As a result, surgeons often adopt cautious approaches in this region, which may lead to suboptimal seizure control [27]. This study addresses this challenge by examining the relationship between PET hypometabolism and postoperative motor function outcomes in patients undergoing Rolandic cortex resection for epilepsy. Through quantitative analysis of PET imaging, we generated SPM-PET score maps that accurately reflect relative metabolic levels. Our previous research demonstrated the reliability and accuracy of this method [28]. Building on this foundation, which included the development and validation of multimodal imaging post-processing techniques, this study integrates fMRI data with PET metabolic analysis [22, 29]. The results of our study suggest that PET hypometabolism may serve as a marker of motor function reorganization. Specifically, we observed that reorganization of hand motor areas tends to involve inferior and lateral displacement from the classical hand-knob region, while lower limb motor areas tend to relocate anteriorly within the paracentral lobule. In the Rolandic area, lower PET metabolism was associated with a reduced likelihood of preserved motor function, indicating that the function may have already undergone distant reorganization. These findings imply that if PET metabolism in the Rolandic cortex falls below a certain threshold, the risk of postoperative motor deficits decreases. Additionally, previous studies have suggested that areas of PET hypometabolism may extend beyond the actual EZ [30], which should be carefully considered when planning the surgical approach. In clinical practice, we observed that a small number of patients with reduced PET metabolism in the Rolandic area did not regain their preoperative muscle strength following surgery. In some cases, their motor function deteriorated, contrary to our initial hypothesis. Further discussion and analysis of postoperative imaging suggested that this outcome may have resulted from inadvertent resection of related pyramidal tracts, which could explain the observed motor deficits.

### The multifaceted role of PET in epilepsy surgery

Previous studies have highlighted the critical role of [^18^F]FDG PET in detecting FCD and precisely localizing the EZ [31]. The impact of various surgical techniques on the prognosis of Rolandic epilepsy surgery has been well documented, particularly regarding the resection of specific areas within the Rolandic cortex and the completeness of FCD resection [32]. Furthermore, postoperative motor function has been shown to strongly correlate with the extent of surgical resection [33]. While most studies have focused on seizure-free outcomes, some have also evaluated the discontinuation of anti-seizure medications (ASMs) and improvements in academic performance and socio-occupational functioning [34]. Beyond surgical outcomes, preoperative evaluation of FCD remains challenging due to the lack of histopathological confirmation at the time clinical decisions are made. In practice, evaluation of FCD typically relies on a comprehensive, multimodal approach that integrates neurophysiological data with structural and metabolic imaging findings. Electrophysiologically, scalp EEG and intracranial EEG provide valuable markers—such as repetitive polyspikes, bursts of fast rhythms, and disruptions of physiological background activity—that are widely recognized as reliable indicators of FCD type II [35]. Structurally, MRI plays a critical role in lesion characterization, with classical features of FCD type II including blurred gray-white matter junctions, increased T2/FLAIR signal intensity (the “transmantle sign”), cortical thickening, and abnormal gyral or sulcal patterns.

Metabolic imaging with [^18^F]FDG PET often reveals focal hypometabolism, further aiding lesion localization, particularly when MRI findings are subtle or inconclusive [36]. Studies have demonstrated that focal PET hypometabolism is strongly associated with FCD type II, especially when presenting with a distinct, unilobar metabolic pattern involving a single gyrus or clearly defined borders. In contrast, such metabolic patterns have not been observed in FCD type I, and PET hypometabolic patterns do not show significant prognostic differences among patients with FCD type I [13]. Additionally, the extent of PET hypometabolism in FCD type II closely corresponds to the epileptogenic zone, underscoring the clinical utility of PET in presurgical evaluation. The anatomical location of lesions also provides critical insights, as certain cortical regions, particularly the frontal lobe, exhibit a higher prevalence of FCD type II. Although histopathological confirmation remains the gold standard for definitive diagnosis, integrating [^18^F]FDG PET findings with electrophysiological and structural imaging data significantly enhances the accuracy and reliability of presurgical evaluation in FCD.

Quantitative analysis of [^18^F]FDG PET facilitates the investigation of metabolic network alterations in patients with different pathological types of epilepsy, emphasizing variations in connectivity across specific brain regions. This analysis offers valuable insights for developing individualized treatment strategies for epilepsy. Furthermore, combining [^18^F]FDG PET with other imaging modalities and electrophysiological data allows for a more comprehensive assessment of the epileptogenic network. Identifying distinct metabolic patterns with FDG-PET can also aid in predicting postoperative outcomes [37]. While [^18^F]FDG PET has traditionally been used to assess glucose consumption patterns in normal brain function, serving as a baseline for early disease diagnosis [38], its role in epilepsy surgery continues to expand. Future studies incorporating advanced neuroimaging analysis techniques, such as radiomics or machine learning, may further improve its predictive value in presurgical evaluation, prognosis assessment, and treatment optimization.

### FCD subtypes

In our study, three patients were pathologically diagnosed with FCD type I; however, their electroclinical and imaging characteristics were more consistent with those typically associated with FCD type II. Consequently, during the presurgical evaluation, these lesions were clinically classified as FCD type II, and focal resections were performed accordingly. We hypothesize that the discrepancy between clinical and pathological classifications could be attributed to the technical challenges of achieving en bloc resection in the Rolandic region, a phenomenon previously discussed in our earlier report [39]. The predictive value of FDG-PET T-values may differ among FCD subtypes. To further assess the predictive capability of PET specifically within FCD type II, we conducted subgroup analyses comparing FCD IIa and IIb cases. In both subgroups, PET metabolic *T*-values were significantly higher in patients experiencing postoperative motor deficits compared to those without deficits. These findings underscore the robustness of PET-based evaluations across FCD type II subtypes, indicating a consistent relationship between PET hypometabolism and clinical motor outcomes.

### Exploring potential mechanisms underlying our findings

Our findings reveal a significant negative correlation between postoperative muscle strength and PET metabolic levels. However, the underlying mechanisms remain unclear. Further research is required to explore potential explanations, such as the role of Betz cell migration, alterations in synaptic connectivity, or the metabolic demands of motor neurons. Betz cells, giant pyramidal neurons concentrated in the primary motor cortex, are known to form monosynaptic connections with brainstem and spinal α-motoneurons [40]. Historically and clinically, Betz cells have been regarded as typical “upper motor neurons” or “pyramidal tract neurons.” Most studies suggest that Betz cells are formed during development and remain fixed in layer V of the primary motor cortex [41]. However, recent research has identified Betz cells in the premotor cortex [42], raising the possibility that these cells may migrate and influence motor function, as evidenced by their presence beyond the primary motor cortex. Moreover, large neuronal somata are indicative of extensive axodendritic trees, which suggest a high metabolic demand. Atrophy, disappearance, or migration of Betz cells in the primary motor cortex would logically result in a corresponding decrease in PET metabolism. Additionally, factors such as the density of afferent and efferent synaptic connections, firing rates, and neurotransmitter types also significantly influence metabolic rates [43]. It is important to recognize that the execution and inhibition of voluntary (planned) movements rely on the activity of complex networks involving various anatomically defined regions within the premotor and primary motor cortices. This process is highly dynamic, requiring continuous interaction among these regions. Further research is needed to better understand these mechanisms. Future studies could focus on detailed investigations of Betz cell distribution, the role of synaptic plasticity in functional reorganization, and the metabolic profiles of affected cortical regions.

### Limitations

We acknowledge several limitations in our study. Although we included 62 patients and conducted statistical analyses on factors such as pathological characteristics, age at onset, duration of epilepsy, and other clinical variables, the relatively small sample size limits our ability to perform detailed stratified analyses that could comprehensively elucidate the impact of these variables. Additionally, while our findings indicate an association between PET hypometabolism and functional reorganization, the precise neurobiological mechanisms underlying this relationship remain unclear. The complexity of cortical reorganization in epilepsy warrants further investigation, particularly using advanced neuroimaging techniques capable of providing deeper insights into the neural pathways involved.

## Conclusions

This study demonstrates a significant positive correlation between PET metabolic levels in the Rolandic cortex and postoperative motor deficits. By integrating fMRI and PET data, we observed that hypometabolism in the classical motor cortex suggests potential cortical motor function reorganization. These findings indicate that in regions with significantly reduced PET metabolism, even within traditionally eloquent cortex areas, the risk of postoperative motor function deficits is relatively low. This study offers a novel perspective on surgical strategies for Rolandic epilepsy, suggesting that more aggressive surgical interventions may be safely pursued in areas of pronounced PET hypometabolism.

## Supplementary Information

Additional File 1: Figures S1-S2. FigS1– [Workflow of Quantitative PET Analysis]. FigS2 – [Logistic regression curve showing the association between the PET T-value in the resected Rolandic cortex and the predicted probability of postoperative motor deficits]

Additional File 2: Table S1 – [Specific patient information]

## Data Availability

The datasets generated during and/or analyzed during the current study are available from the corresponding author on reasonable request.

## Abbreviations

ASMs: Anti-seizure medications
AUC: Area under the curve
CI: Confidence intervals
DTI: Diffusion tensor imaging
EZ: Epileptogenic zone
FCD: Focal cortical dysplasia
[^18^F]FDG PET: ^18^F-fluorodeoxyglucose positron emission tomography
fMRI: Functional magnetic resonance imaging
FOV: Field of view
FWHM: Full-width at half-maximum
MEG: Magnetoencephalography
MEP: Motor evoked potentials
OR: Odds ratios
ROI: Regions of interests
SEEG: Stereoelectroencephalography
TE: Echo time
TR: Repetition time
