# Supplementary material for: Predicting postoperative motor outcomes in the surgical management of Rolandic focal cortical dysplasia: the role of glucose metabolism
Source: BMC Med. 2025 Jul 1;23:390. doi: 10.1186/s12916-025-04213-9 (PMC12220331; doi:10.1186/s12916-025-04213-9)
Supplement: Supplementary file 1 — Additional File 1: Figures S1-S2. FigS1– [Workflow of Quantitative PET Analysis]. FigS2 – [Logistic regression curve showing the association between the PET T-value in the resected Rolandic cortex and the predicted probability of postoperative motor deficits] [file 12916_2025_4213_MOESM1_ESM.docx]

**Additional files 1 - figure of content**

[**Fig. S1** **Workflow of Quantitative PET Analysis. 2**](file:///C:\Users\DELL\Downloads\12916_2025_3897_MOESM2_ESM.docx#_Toc23558)

[**Fig. S2** **Logistic regression curve showing the association between the PET T-value in the resected Rolandic cortex and the predicted probability of postoperative motor deficits. 3**](file:///C:\Users\DELL\Downloads\12916_2025_3897_MOESM2_ESM.docx#_Toc19753)


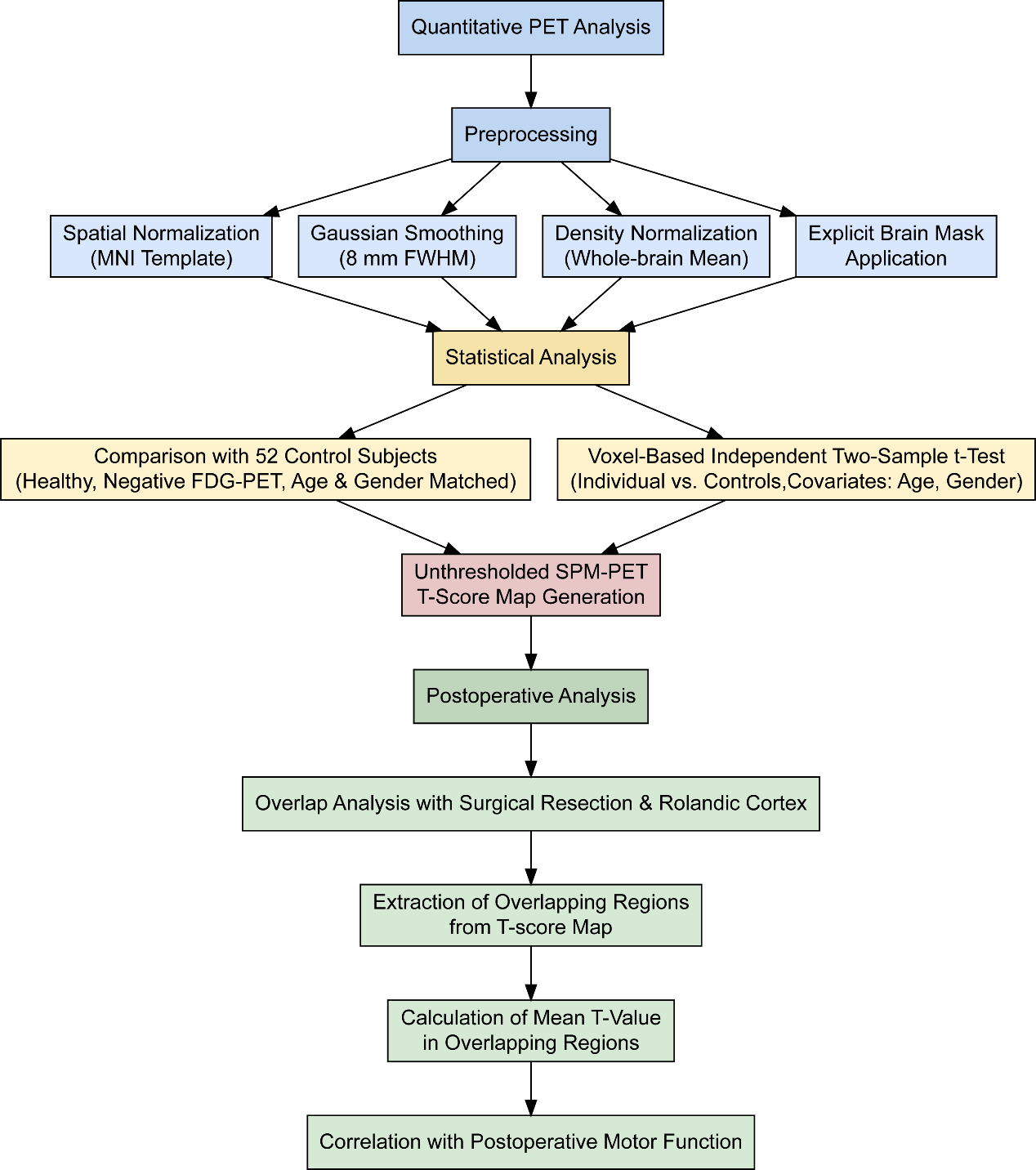


**Fig. S1** Workflow of Quantitative PET Analysis


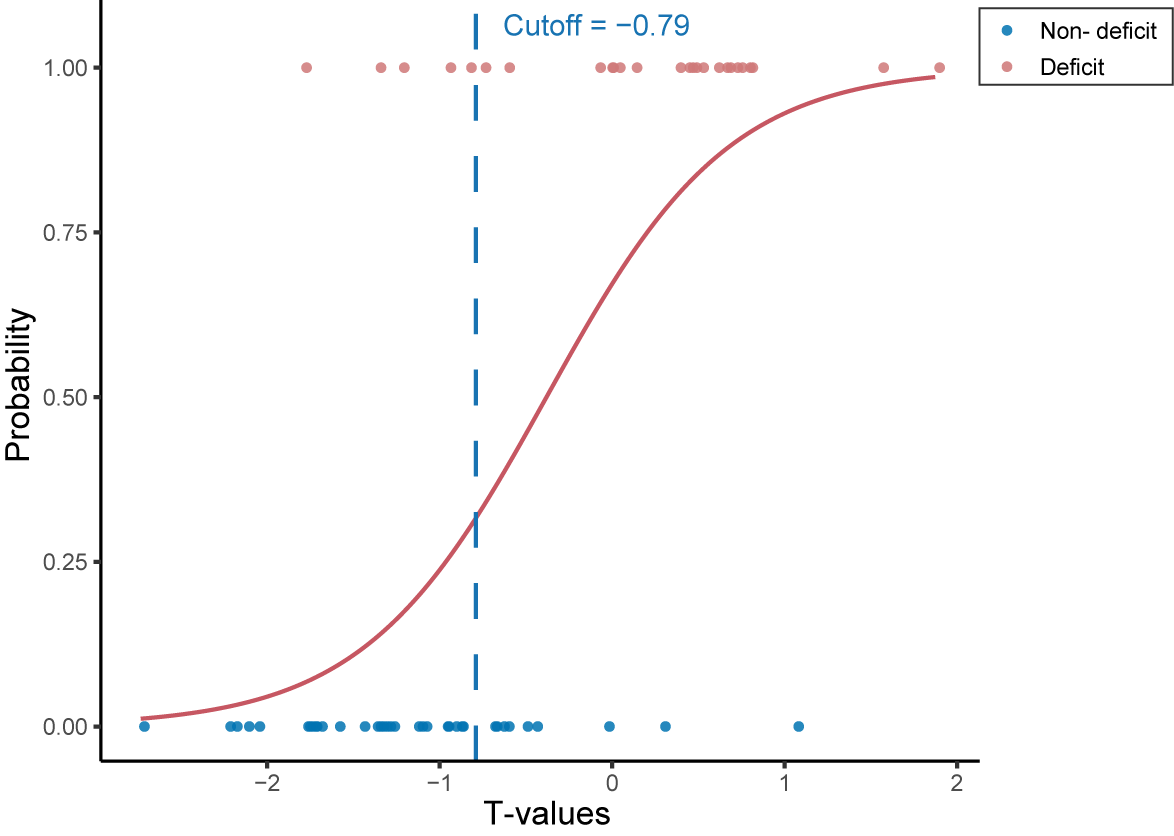


**Fig. S2** Logistic regression curve showing the association between the PET T-value in the resected Rolandic cortex and the predicted probability of postoperative motor deficits. Each dot represents a single patient (blue: no deficit; red: motor deficit). The red curve indicates the model-predicted probability of motor impairment. The dashed vertical line (T = –0.79) marks the optimal cutoff derived from ROC analysis. Higher T-values were significantly associated with increased risk of postoperative motor deficits (OR = 8.67, 95% CI: 2.77–25.63).
