# Supplementary material for: Predicting postoperative motor outcomes in the surgical management of Rolandic focal cortical dysplasia: the role of glucose metabolism
Source: BMC Med. 2025 Jul 1;23:390. doi: 10.1186/s12916-025-04213-9 (PMC12220331; doi:10.1186/s12916-025-04213-9)
Supplement: Supplementary file 2 — Additional File 2: Table S1 – [Specific patient information] [file 12916_2025_4213_MOESM2_ESM.docx]

Additional files 2 - table of content

[Table S1. Specific patient information 2](#_Toc4311)

**Table S1. Specific patient information**

| ID | Gender | Age | Duration  (year) | Onset | Follow-up  (year) | Side | Deficit | Strength | Lesion | Path | MRI |
| --- | --- | --- | --- | --- | --- | --- | --- | --- | --- | --- | --- |
| 1 | M | 25 | 17 | 8 | 2 | L | Y | Ⅴ | L.Fr | Ⅱa | - |
| 2 | M | 16 | 19 | 7 | 3 | R | N | Ⅴ | R.Fr | Ⅱa | - |
| 3 | M | 28 | 29 | 4 | 3 | L | Y | Ⅱ | L.Fr | Ⅱa | - |
| 4 | F | 7 | 21 | 6 | 2 | R | N | Ⅴ | R.Fr | Ⅱa | - |
| 5 | M | 26 | 25 | 4 | 6 | R | Y | Ⅳ | R.Fr | Ⅱa | - |
| 6 | M | 33 | 33 | 5 | 3 | R | Y | Ⅲ | R.Fr | Ⅱa | - |
| 7 | F | 27 | 3 | 2 | 5 | L | Y | Ⅳ | L.Fr | Ⅱa | - |
| 8 | M | 29 | 4 | 6 | 4 | L | N | Ⅴ | L.P | Ⅱb | - |
| 9 | M | 38 | 4 | 3 | 4 | L | N | Ⅴ | L.P | Ⅰb | - |
| 10 | F | 5 | 15 | 10 | 1 | R | Y | Ⅳ | R.FP | Ⅱb | - |
| 11 | M | 12 | 6 | 6 | 3 | R | Y | Ⅳ | R.Fr | Ⅱa | - |
| 12 | M | 7 | 1 | 5 | 3 | R | Y | Ⅳ | R.P | Ⅱb | - |
| 13 | M | 31 | 7 | 12 | 3 | L | N | Ⅴ | L.P | Ⅱa | - |
| 14 | F | 25 | 13 | 9 | 2 | L | N | Ⅴ | L.P | Ⅱb | - |
| 15 | F | 12 | 1 | 4 | 4 | R | N | Ⅴ | R.Fr | Ⅱa | - |
| 16 | M | 16 | 7 | 17 | 2 | R | N | Ⅴ | R.P | Ⅱa | - |
| 17 | F | 6 | 10 | 8 | 6 | L | Y | Ⅱ | L.FP | Ⅱb | - |
| 18 | M | 19 | 12 | 1 | 1 | R | N | Ⅴ | R.Fr | Ⅱa | - |
| 19 | F | 22 | 9 | 5 | 2 | R | Y | Ⅳ | R.Fr | Ⅱb | - |
| 20 | F | 8 | 4 | 4 | 6 | R | Y | Ⅳ | R.Fr | Ⅱa | - |
| 21 | F | 5 | 1.5 | 7.5 | 2 | R | Y | Ⅳ | R.Fr | Ⅱa | - |
| 22 | F | 24 | 4 | 6 | 3 | R | N | Ⅴ | R.Fr | Ⅱa | - |
| 23 | M | 18 | 11 | 4 | 2 | L | Y | Ⅳ | L.Fr | Ⅱa | - |
| 24 | M | 13 | 1.5 | 5.5 | 2 | L | N | Ⅴ | L.P | Ⅰa | - |
| 25 | F | 14 | 1.75 | 1.2 | 6 | R | N | Ⅴ | R.Fr | Ⅱb | - |
| 26 | M | 8 | 22 | 10 | 2 | R | N | Ⅴ | L.Fr | Ⅱa | - |
| 27 | M | 9 | 21 | 6 | 2 | L | N | Ⅴ | R.Fr | Ⅱa | - |
| 28 | F | 10 | 12 | 5 | 5 | R | N | Ⅴ | R.Fr | Ⅱb | - |
| 29 | M | 15 | 1.8 | 5.2 | 3 | L | Y | Ⅱ | L.FP | Ⅱa | - |
| 30 | M | 7 | 25 | 12 | 4 | L | Y | Ⅲ | L.FP | Ⅱa | - |
| 31 | M | 3 | 1.5 | 11.5 | 2 | L | Y | Ⅳ | L.Fr | Ⅰb | - |
| 32 | M | 27 | 10 | 13 | 7 | R | N | Ⅴ | R.P | Ⅱb | - |
| 33 | M | 27 | 4 | 16 | 3 | L | N | Ⅴ | L.Fr | Ⅱa | - |
| 34 | M | 13 | 4 | 9 | 2 | L | Y | Ⅳ | L.Fr | Ⅱa | - |
| 35 | M | 17 | 12 | 5 | 5 | R | N | Ⅴ | R.Fr | Ⅱb | - |
| 36 | F | 44 | 40 | 4 | 3 | R | N | Ⅴ | R.Fr | Ⅱa | - |
| 37 | F | 7 | 1.8 | 5.2 | 3 | L | N | Ⅴ | L.FP | Ⅱb | - |
| 38 | M | 37 | 25 | 12 | 4 | L | Y | Ⅲ | L.Fr | Ⅱa | - |
| 39 | F | 13 | 1.5 | 11.5 | 5 | R | Y | Ⅲ | R.P | Ⅱa | - |
| 40 | F | 23 | 10 | 13 | 6 | R | N | Ⅴ | R.P | Ⅱb | - |
| 41 | M | 20 | 4 | 16 | 5 | L | N | Ⅴ | L.Fr | Ⅱa | - |
| 42 | F | 3.3 | 3 | 0.3 | 7 | L | N | Ⅴ | L.Fr | Ⅱb | + |
| 43 | M | 32 | 26 | 6 | 3 | R | N | Ⅴ | R.Fr | Ⅱa | + |
| 44 | F | 8 | 6 | 2 | 7 | R | Y | Ⅳ | R.P | Ⅱb | + |
| 45 | M | 2.2 | 1.8 | 0.4 | 1 | L | N | Ⅴ | L.Fr | Ⅱa | + |
| 46 | F | 4 | 0.1 | 3.9 | 1 | R | N | Ⅴ | R.Fr | Ⅱa | + |
| 47 | F | 16 | 9 | 7 | 4 | L | N | Ⅴ | L.Fr | Ⅱb | + |
| 48 | F | 2 | 1.3 | 0.7 | 2 | R | Y | Ⅱ | R.Fr | Ⅱb | + |
| 49 | M | 8 | 7.9 | 0.1 | 5 | R | N | Ⅴ | R.P | Ⅱa | + |
| 50 | F | 5 | 0.87 | 4.13 | 4 | R | N | Ⅴ | R.Fr | Ⅱb | + |
| 51 | F | 10 | 9 | 1 | 3 | R | Y | Ⅱ | R.Fr | Ⅱa | + |
| 52 | F | 5.5 | 4 | 1.5 | 8 | R | Y | Ⅲ | L.Fr | Ⅱb | + |
| 53 | F | 5 | 1 | 4 | 6 | L | N | Ⅴ | L.Fr | Ⅱa | + |
| 54 | F | 7 | 0.5 | 6.5 | 3 | R | Y | Ⅳ | R.Fr | Ⅱa | + |
| 55 | F | 3 | 2 | 1 | 1 | R | N | Ⅴ | R.Fr | Ⅱa | + |
| 56 | F | 14 | 8 | 6 | 3 | R | N | Ⅴ | R.Fr | Ⅱb | + |
| 57 | M | 26 | 25 | 1 | 1 | L | Y | Ⅲ | L.P | Ⅱa | + |
| 58 | F | 15 | 6 | 9 | 2 | L | N | Ⅴ | L.P | Ⅱa | + |
| 59 | M | 13 | 7 | 6 | 1 | L | Y | Ⅳ | L.Fr | Ⅱb | + |
| 60 | M | 12 | 0.25 | 12 | 7 | L | N | Ⅴ | L.Fr | Ⅱb | + |
| 61 | M | 9 | 0.75 | 8.25 | 7 | R | Y | Ⅳ | R.P | Ⅱb | + |
| 62 | M | 6 | 2.3 | 3.7 | 6 | R | N | Ⅴ | R.Fr | Ⅱb | + |

+: positive; -: negative; F: female; FP: frontoparietal; Fr: frontal; L: left; M: male; R: right; Path: Pathology; Y: Yes; N: No
